# The KSHV ORF20 Protein Interacts with the Viral Processivity Factor ORF59 and Promotes Viral Reactivation

**DOI:** 10.1128/spectrum.00145-21

**Published:** 2021-06-09

**Authors:** D. Hoffman, W. Rodriguez, D. Macveigh-Fierro, J. Miles, M. Muller

**Affiliations:** a Microbiology Department, University of Massachusetts Amherst, Amherst, Massachusetts, USA; Broad Institute

**Keywords:** DNA replication, Kaposi's sarcoma-associated herpesvirus, herpesviruses

## Abstract

Upon Kaposi’s Sarcoma-associated herpesvirus (KSHV) lytic reactivation, rapid and widespread amplification of viral DNA (vDNA) triggers significant nuclear reorganization. As part of this striking shift in nuclear architecture, viral replication compartments are formed as sites of lytic vDNA production along with remarkable spatial remodeling and the relocalization of cellular and viral proteins. These viral replication compartments house several lytic gene products that coordinate viral gene expression, vDNA replication, and nucleocapsid assembly. The viral proteins and mechanisms that regulate this overhaul of the nuclear landscape during KSHV replication remain largely unknown. KSHV’s *ORF20* is a widely conserved lytic gene among all herpesviruses, suggesting it may have a fundamental contribution to the progression of herpesviral infection. Here, we utilized a promiscuous biotin ligase proximity labeling method to identify the proximal interactome of ORF20, which includes several replication-associated viral proteins, one of which is ORF59, the KSHV DNA processivity factor. Using coimmunoprecipitation and immunofluorescence assays, we confirmed the interaction between ORF20 and ORF59 and tracked the localization of both proteins to KSHV replication compartments. To further characterize the function of ORF20, we generated an ORF20-deficient KSHV and compared its replicative fitness to that of wild-type virus. Virion production was significantly diminished in the ORF20-deficient virus as observed by supernatant transfer assays. Additionally, we tied this defect in viable virion formation to a reduction in viral late gene expression. Lastly, we observed an overall reduction in vDNA replication in the ORF20-deficient virus, implying a key role for ORF20 in the regulation of lytic replication. Taken together, these results capture the essential role of KSHV ORF20 in progressing viral lytic infection by regulating vDNA replication alongside other crucial lytic proteins within KSHV replication compartments.

**IMPORTANCE** Kaposi’s Sarcoma-associated herpesvirus (KSHV) is a herpesvirus that induces lifelong infection, and as such, its lytic replication is carefully controlled to allow for efficient dissemination from its long-term reservoir and for the spread of the virus to new hosts. Viral DNA replication involves many host and viral proteins, coordinating both in time and space to successfully progress through the viral life cycle. Yet, this process is still not fully understood. We investigated the role of the poorly characterized viral protein ORF20, and through proximity labeling, we found that ORF20 interacts with ORF59 in replication compartments and affects DNA replication and subsequent steps of the late viral life cycle. Collectively, these results provide insights into the possible contribution of ORF20 to the complex lytic DNA replication process and suggest that this highly conserved protein may be an important modulator of this key viral mechanism.

## INTRODUCTION

Human herpesvirus 8, also known as Kaposi’s Sarcoma-associated herpesvirus (KSHV), is an oncogenic virus that has the incredible ability of initiating and maintaining lifelong infections in its host (1). In immunocompromised individuals, these lifelong infections can lead to the development of several malignancies, including Kaposi’s sarcoma (KS), primary effusion lymphoma (PEL), and multicentric Castleman disease (MCD) (2–4). Similar to other herpesviruses, KSHV undergoes a biphasic life cycle that features both latent and lytic phases (1). During latency, KSHV can persist for decades within its infected host, during which time only a few viral proteins are expressed. These latency-associated proteins in concert with host machinery coordinate viral genome maintenance and replication while evading detection by host immune surveillance. However, upon reactivation into the lytic phase, the vast majority of KSHV genes are expressed in an ordered cascade, rapidly amplifying the viral genome and triggering the assembly of new viral particles. As with other herpesviruses, KSHV lytic phase replication progresses via a mechanism distinct from that of latent viral DNA replication (5). In particular, lytic replication initiates at a different origin of replication (ori-Lyt) on the viral genome and does not occur in synchrony with the host cell (6, 7). Instead, KSHV lytic replication requires at least eight viral proteins (ORF9, ORF6, ORF40, ORF44, ORF56, ORF50, ORF59, and ORFK8), including its own virally encoded DNA polymerase (ORF9) (1, 8, 9) and DNA polymerase processivity factor (ORF59) (10–12). In particular, ORF59 has been shown to be recruited to the lytic origin of replication via its binding to ORF50 (RTA) (13), which leads to the recruitment of the viral DNA polymerase and initiation of replication (10, 11, 14). Therefore, KSHV lytic DNA replication is reliant on the recruitment of ORF59 and the assembly of a replication complex composed of multiple viral lytic factors.

To accommodate the large-scale amplification of the viral genome, lytic replication is accompanied by a drastic reorganization of the nucleus. This results in major nuclear and nucleolar remodeling, including relocalization of host chromatin and displacement of several host proteins such as nucleolin and nucleophosmin (15). This represents one of the hallmarks of herpesvirus replication: the formation of a “bean-shaped” viral replication compartment (RC) (16–19).

Among the ∼85 viral proteins encoded by the KSHV genome, ORF20 belongs to the herpesviral core *UL24* gene family, widely conserved throughout the *Alpha*-, *Beta*-, and *Gammaherpesvirinae* (20, 21). Very little is known about the functions of KSHV ORF20 and its orthologs across the herpesvirus family. For ORF20 orthologs harbored by HSV-1 (UL24), human cytomegalovirus (HCMV) (UL76), and murine gammaherpesvirus 68 (MHV68) (ORF20), each have been reported to modulate cell cycle arrest and apoptosis (22–24). Past studies have also shown that KSHV ORF20 and its orthologs UL24 and UL76 localize to the nucleoli of transfected cells (25–27) with a demonstrably complex localization pattern (28). Furthermore, UL24 was shown to contribute to nucleolar reorganization by affecting the expression pattern of key nucleolar proteins (25, 29–31). ORF20 mRNA expression kinetics seem to vary depending on the host cell, demonstrating late gene kinetics upon *de novo* infection of primary human umbilical vein endothelial cells (HUVEC) and upon reactivation of a latently infected body cavity-based lymphoma cell line (BCBL1) (32). However, more recently, upon lytic reactivation in endothelial cells, KSHV *ORF20* was shown to be expressed as an immediate early viral gene (28). It was also demonstrated that through its interaction with oligoadenylate synthetase-like protein (OASL), ORF20 enhances KSHV replication, possibly by regulating ribosome composition (28). Given that ORF20 is widely conserved in the herpesvirus family and based on these roles gathered from several herpesvirus members, we set out to investigate the role of ORF20 in KSHV and whether its function could bridge these observations of both nuclear remodeling and high conservation.

Here, we investigated the ORF20 microenvironment by proximity labeling using BioID and identified ORF59 as an ORF20 interactor. We then tracked the localization of ORF20 upon lytic reactivation and found it localizes to KSHV replication compartments, where it colocalizes with ORF59. Furthermore, we found that cells infected with an ORF20-deficient virus have a severe defect in viral DNA replication, late gene expression, and subsequent virion formation. Taken together, our results indicate that ORF20 is an important regulator of the KSHV lytic cycle. By placing ORF20 at the replication compartment where it interacts with KSHV processivity factor ORF59, our work supports a role for ORF20 in KSHV complex lytic DNA replication.

## RESULTS

### KSHV ORF20 proximal proteome identifies novel ORF20 interactors.

**FIG 1:**
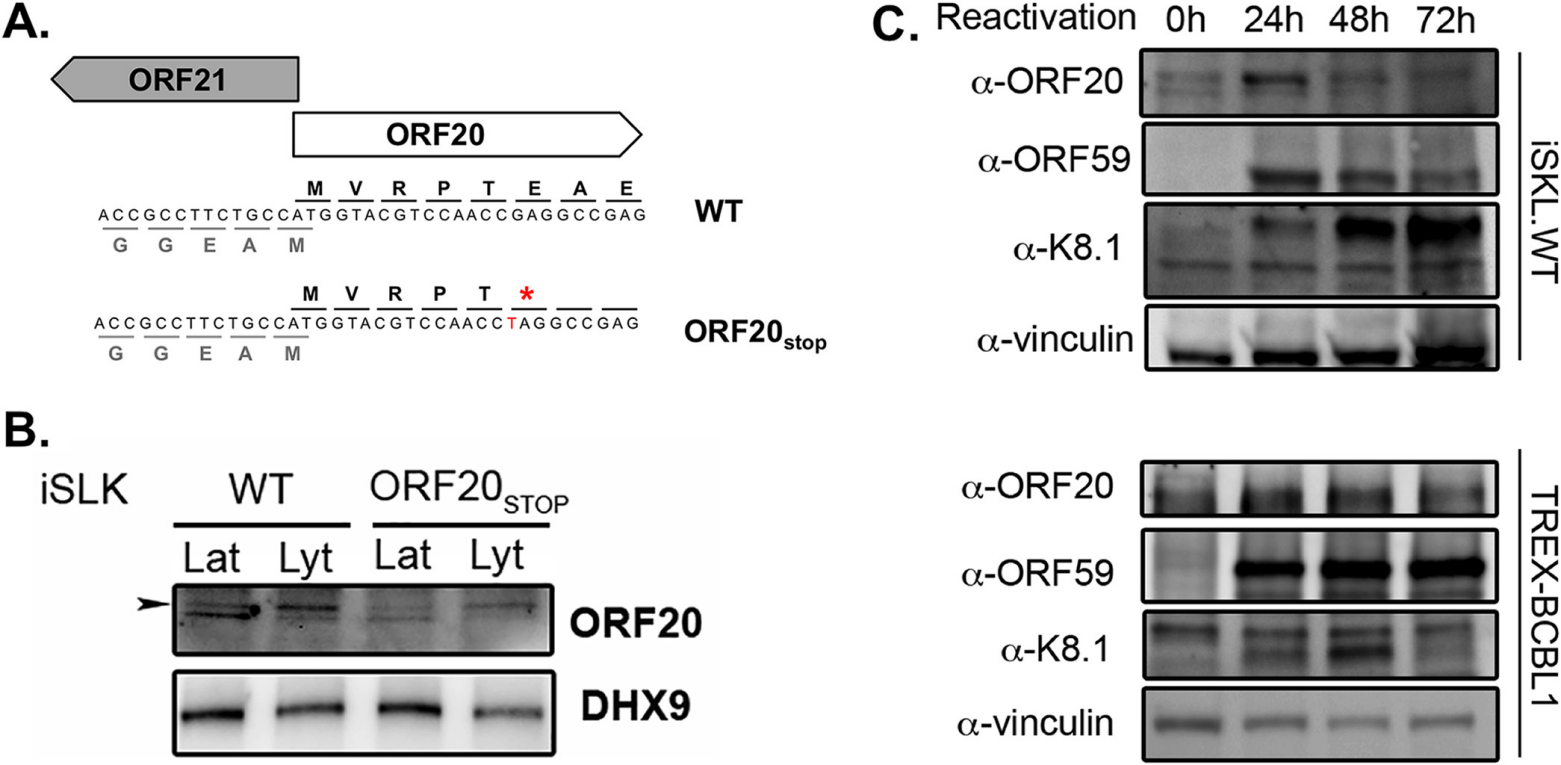
Characterization of the expression of KSHV ORF20. (A) Diagram showing the genomic locus of *ORF20*, with surrounding gene *ORF21*, depicting the location of the introduced mutation. (B) ORF20 expression was assessed by Western blotting in both iSLK.WT and ORF20STOP cells whether in their latent phase (Lat) or lytic phase (Lyt). DHX9 served as a loading control. (C) Western blot showing the expression kinetics of ORF20 in iSLK.WT cells (top) and TREX-BCBL1 cells (bottom) after reactivation with doxycycline and sodium butyrate for the indicated times. ORF59 (early kinetic) and K8.1 (late kinetic) expression is shown as controls. Vinculin served as a loading control.

To better understand the function of ORF20 during lytic KSHV infection, we used the proximity labeling method BioID to characterize the ORF20 microenvironment. We first generated an ORF20-deficient virus (ORF20_STOP_) using the BAC16 Red recombinase system (33) by mutating the *ORF20* genomic sequence 16 nucleotides downstream of its translation start site (Fig. 1A). This mutation resulted in the expression of a premature termination codon. This virus was used to establish a latently infected cell line in iSLK cells that are here referred to as iSLK-ORF20_STOP_. Using a newly generated ORF20 antibody, we confirmed that ORF20 expression is abrogated in these cells (Fig. 1B). Because the timing of ORF20 expression is crucial to set our experimental timeline, and due to expression being previously inconsistent, we next tested when endogenous ORF20 was made in iSLK.WT cells, a KSHV-positive cell line similar to the one used to establish our ORF20-deficient viral mutant. Based on observations of ORF20 expression in iSLK.WT cells relative to that in the ORF20_STOP_ cells, the band corresponding to ORF20 is the upper band in the doublet detected with this antibody (Fig. 1B). ORF20 was previously shown to express several isoforms, it is thus possible that these band doublets detected here represent two different isoforms. We further explored the kinetic expression of ORF20 in these cells and detected a peak of ORF20 expression as soon as 24 h post-lytic reactivation (Fig. 1C). This is similar to the expression of ORF59, a KSHV early gene, but much earlier than K8.1, a KSHV late gene. Based on this pattern, ORF20 can be considered an early gene in these cells as was recently reported (28). We also investigated its expression in TREX-BCBL1 cells, a KSHV-positive B cell line. In these cells, expression of ORF20 was faint, and we no longer detected the doublet observed in iSLK cells, possibly suggesting that only one isoform is expressed in these cells. Here, we also detected expression at 24 h postreactivation; however, as opposed to that in iSLK cells, its expression was stable at 48 h, highlighting a possible cell-specific regulation of ORF20 expression.

**FIG 2:**
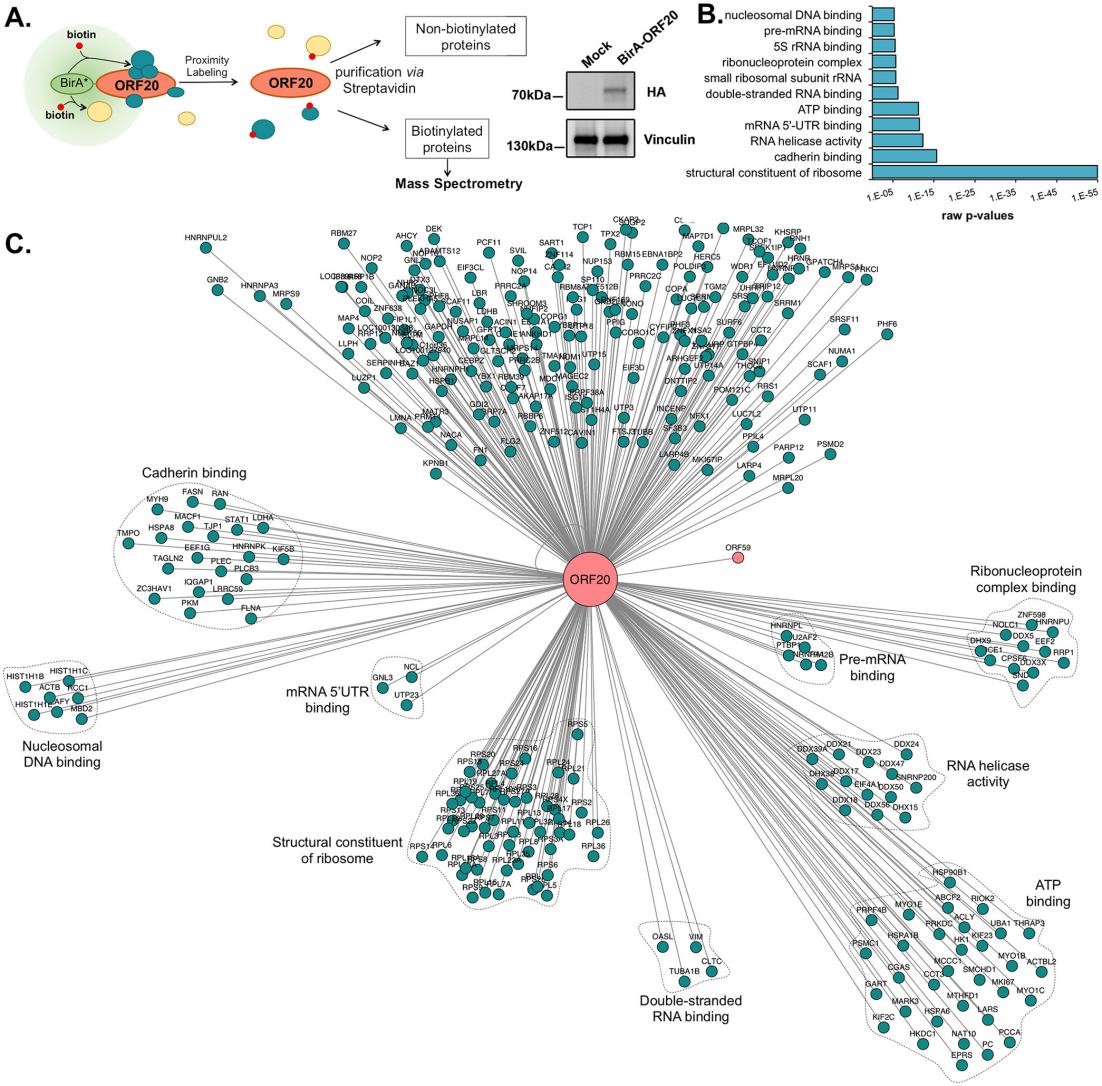
Determination of ORF20 proximal interactome. (A) Schematic representation of the BioID assay, where ORF20 is fused to the biotin ligase BirA which will transfer a biotin to proteins coming in close proximity to ORF20. Expression of the BirA-ORF20 fusion is shown on the right. Vinculin serves as a loading control (B) iSLK.ORF20_STOP_ cells were transfected with ORF20-BirA and subjected to BioID and mass spectrometry. The bar graph represents the top GO terms associated with ORF20 interacting partners, and the network, generated by Cytoscape, represents the ORF20 proximal interactome. Proteins annotated in the categories highlighted in the bar graph are shown in clusters.

To investigate the ORF20 proximal interactome, we used the proximity labeling method BioID in which a promiscuous biotin ligase—BirA—is fused to the protein of interest. Biotinylated proteins resulting from the action of this BirA ligase can then be selectively isolated and identified by mass spectrometry (34). We thus generated a plasmid in which the BirA_R118G_ biotin ligase was fused at the C terminus of ORF20, which was subsequently used to rescue ORF20 expression in the iSLK-ORF20_STOP_ cells. This setup was used to identify novel ORF20 direct and indirect interactors. iSLK-ORF20_STOP_ cells were reactivated for 24 h with doxycycline and sodium butyrate to trigger KSHV lytic cycle and transfected with BirA-ORF20 (or mock transfected) with excess biotin added to the medium at the time of transfection. After 16 h, cells were lysed, and streptavidin-conjugated beads were used to isolate biotinylated proteins. After verifying the expression of our BirA-ORF20-hemagglutinin (HA) construct by Western blotting, the identity of the biotinylated proteins was determined by liquid chromatography-tandem mass spectrometry (LC-MS/MS) (Fig. 2A). As shown in Table S1 in the supplemental material, after filtering, we identified 316 high-confidence unique proteins in the ORF20 microenvironment, of which, 62 were previously identified with KSHV ORF20 (28). Based on gene ontology (GO) term analysis on ORF20 interactors, several functional categories emerged, including several related to ribosomal regulation, as previously reported (Fig. 2B). Intriguingly, several categories placed ORF20 at the nucleosome and close to proteins regulating helicase activity. Moreover, we detected ORF59, the KSHV lytic DNA processivity factor, which we decided to explore further.

### KSHV ORF20 interacts with ORF59 and localizes to the replication compartment.

**FIG 3:**
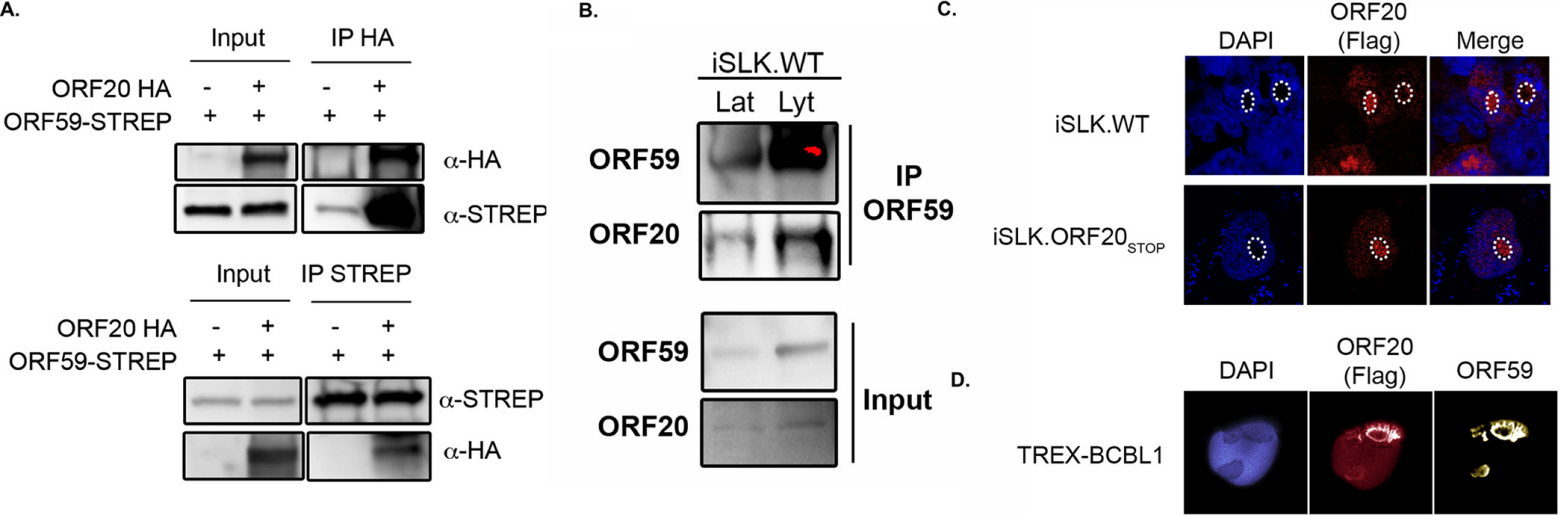
ORF20 interacts and colocalizes with ORF59 in KSHV replication compartments. (A) HEK293T cells were transiently transfected with HA-ORF20 (or mock) and streptavidin (strep)-ORF59 as indicated, and a co-IP was performed using anti-HA antibody. Reverse Co-IP was performed using anti-strep antibody. (B) Lysates of latently infected (Lat) or doxycycline (DOX)-reactivated (Lyt) KSHV-positive iSLK.WT cells were subjected to IP with an anti-ORF59 antibody, and then Western blotted (WB) for ORF20 or ORF59. (C) Reactivated KSHV-positive iSLK.WT or iSLK.ORF20_STOP_ cells were transfected with Flag-ORF20 and, 24 h later, were subjected to immunofluorescence assay using an anti-Flag antibody (red) and DAPI staining to identify nuclei (blue). White dotted circles denote DAPI-deficient nuclear domain representing KSHV replication compartments. (D) DOX-, TPA-, and ionomycin (iono)-treated KSHV-positive TREX-BCBL1 cells were transfected with Flag-ORF20 and, 24 h, later subjected to immunofluorescence assay using an anti-Flag antibody (red), anti-ORF59 antibody (yellow), and DAPI staining to identify nuclei (blue).

To validate the interaction between ORF20 and ORF59 detected by BioID, we used coimmunoprecipitation (Co-IP). We first coexpressed ORF20 and ORF59 in HEK293T cells and confirmed the interaction observed in our BirA experiment (Fig. 3A). Furthermore, we tested the interaction directly in the KSHV-positive cell line iSLK.WT. Cell lysates were collected from iSLK.WT cells in either their latent or reactivated state when both ORF59 and ORF20 should be expressed. We immunoprecipitated ORF59 overnight and immunoblotted the eluates. We found that ORF59 and ORF20 interact during KSHV lytic phase (Fig. 3B).

ORF59 has a distinctive subcellular localization during KSHV lytic cycle and localizes to replication compartments. These replication compartments are the site of the viral genome replication and are easily identifiable as dark areas in 4′,6-diamidino-2-phenylindole (DAPI) staining of host nuclei. Given that ORF20 was previously shown to localize to discrete nuclear foci, we hypothesized that ORF20 could colocalize with ORF59 at these RCs. We first investigated if we could detect a fraction of ORF20 in the RC in reactivated iSLK cells by immunofluorescence assay (IFA). In iSLK.WT cells transfected with Flag-ORF20, ORF20 expression was predominantly nuclear and distinctively present in nuclear puncta overlapping DAPI-negative areas. Because, in iSLK.WT cells, endogenous viral ORF20 expression may interfere with our Flag-tagged construct, we confirmed this expression pattern in iSLK.ORF20_STOP_ cells, where we also found ORF20 expressed in DAPI-negative areas of the nucleus (Fig. 3C). Since this expression pattern is consistent with ORF59 localization patterns and our BioID and Co-IP indicate an interaction between ORF59 and ORF20, we next monitored the localization of both ORF59 and ORF20. To do so, we used the KSHV-positive TREX-BCBL1 cells, since they do not have any fluorescent marker and thus allowed us to use an additional color channel. In these cells, ORF59 and ORF20 appear to colocalize in the nucleus in areas reminiscent of replication compartments (Fig. 3C).

### KSHV lacking ORF20 shows defects in virion formation and late gene expression.

**FIG 4:**
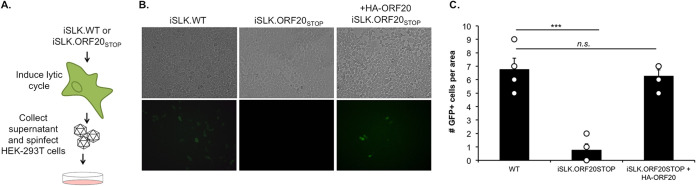
Lack of ORF20 reduces virion production. (A) Diagram depicting the supernatant transfer assay in iSLK cells. (B) Supernatant transfer assay was used as a proxy for virion production and performed as described in panel A. Infection of live HEK293T cells was monitored by contrast (top) and green fluorescent protein (GFP) imaging (bottom) on a fluorescence microscope. (C) Quantification of GFP-positive cells using ImageJ. Values represent four independent views of the infected cells.

Given that ORF20 function is poorly understood, we next wanted to better define its role during the KSHV lytic cycle. Based on our data, ORF20 is expressed early in the lytic cycle and is expressed in RCs where it interacts with ORF59. Moreover, ORF20 is very well conserved in the herpesvirus family, suggesting that its function might be crucial for proper progression of viral infection, and so we hypothesized that ORF20 could be important for the regulation of viral replication. To address this question, we first monitored the ability of the iSLK.ORF20_STOP_ cells to produce progeny virions by supernatant transfer assay and showed that, compared to iSLK.WT cells, these almost completely failed to produce any viable virions (Fig. 4B and C). When ORF20 expression was rescued using ectopic expression of our HA-tagged ORF20 plasmid, virion production was restored, suggesting that the defect observed in these cells is caused by the lack of ORF20 expression.

**FIG 5:**
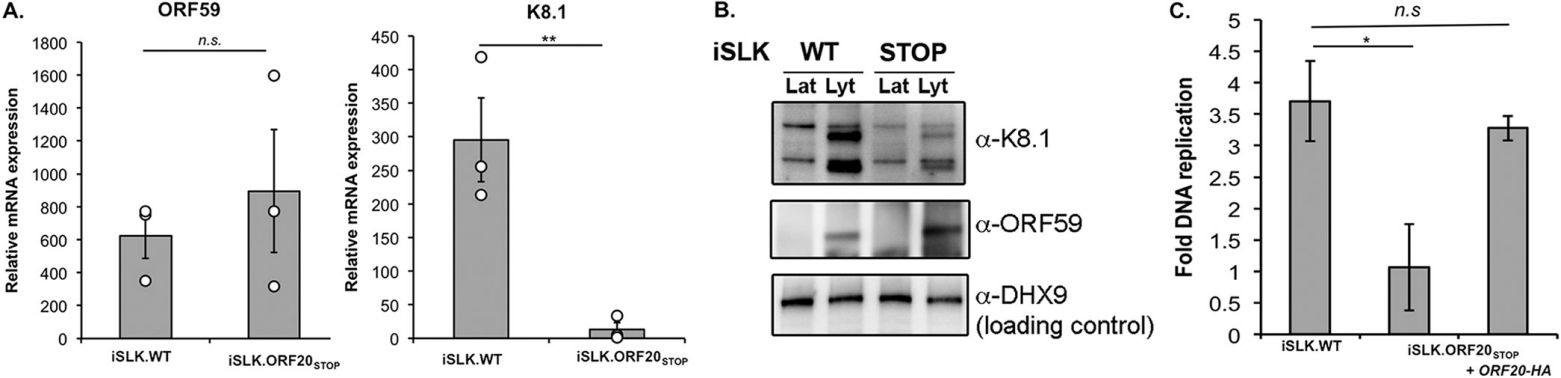
ORF20 is involved in regulating DNA replication and subsequent late gene expression. (A and B) Total RNA was extracted from unreactivated and reactivated iSLK.WT or iSLK.ORF20_STOP_ cells. (A) RNA was then subjected to RT-qPCR to quantify expression of the indicated viral genes. Bars represent the relative mRNA fold change over that in unreactivated cells. (B) Western blot showing the expression of ORF59 and K8.1 in iSLK.WT or iSLK.ORF20_STOP_ cells after reactivation with doxycycline and sodium butyrate. DHX9 served as a loading control. (C) DNA replication was measured by qPCR of the viral genome before and after reactivation of the lytic cycle iSLK.WT or iSLK.ORF20_STOP_ cells. ORF20 expression was complemented by exogenous expression of ORF20-HA (right bar).

Provided that the ORF20_STOP_ cells were severely deficient in virion production, we next investigated whether ORF20 expression, or lack thereof, influences viral gene expression. We investigated the expression kinetics of two viral genes for which we have antibodies: the early viral protein ORF59 and the late major capsid protein K8.1. iSLK.WT or iSLK.ORF20_STOP_ cells were reactivated, and either total RNA was extracted and used for reverse-transcription quantitative PCR (RT-qPCR) or cell lysates were collected and used for immunoblotting. Compared with iSLK.WT cells, iSLK.ORF20_STOP_ cells displayed a drastic reduction of K8.1 expression both at the RNA (Fig. 5A) and protein (Fig. 5B) levels but only a marginal effect on ORF59. Because of this differential effect on early (ORF59) versus late (K8.1) gene expression and because ORF20 appears to be expressed in replication compartments, we hypothesized that ORF20 could have an impact on KSHV DNA replication. Therefore, we next quantified this process in both iSLK.WT cells and iSLK.ORF20_STOP_ cells. We observed a reduction in viral DNA replication in cells lacking ORF20 expression, which was rescued by ectopic expression of ORF20 (Fig. 5C). This suggests that the presence of ORF20 is required for proper progression of KSHV DNA replication and, consequently, late gene expression and viable virion production.

## DISCUSSION

In this study, we demonstrate that KSHV ORF20 localizes to replication compartments where it interacts with KSHV DNA replication processivity factor ORF59. Our approach to better define the contribution of ORF20 to KSHV lytic replication was to investigate the ORF20 microenvironment by proximity labeling. Namely, we used BioID to uncover ORF20 proximal interacting partners, complementing past work conducted to reveal the interactome of ORF20 (28). We confirmed many of the previously identified ORF20 protein partners, including many ribosomal factors as well as OASL, as was previously reported (28). The interaction between ORF20 and OASL has actually been extensively characterized, and given the known roles of OASL in interferon regulation and antiviral immunity, it would be interesting to investigate whether its interaction with ORF20 affects the viral life cycle that we detected in this study (35). In addition, our approach includes not only direct interaction but also transient interactions and a close proximity factor and therefore greatly expands the scope of ORF20 potential partners.

A GO term analysis of the ORF20 microenvironment also revealed several novel putative functions for ORF20, with many relating to RNA binding. Since ORF20 was previously hypothesized to help bridge specific mRNAs to polysomes (28), it would be interesting to further investigate this aspect of ORF20 biology. In addition, nucleolin (NCL) was identified to be in close proximity to ORF20, which is intriguing, since ORF20 ortholog UL24 in HSV-1 was shown to be directly involved in the dispersal of NCL during infection (29). This suggests that this dispersal, which is also observed during KSHV infection (36, 37), might be a conserved function of ORF20.

We also show that ORF20 has a distinctive subcellular localization that we identified is in replication compartments. Of note, we also noticed that ORF20 subcellular localization was heterogeneous, and we observed that ORF20 could also be more diffuse in the nucleus dependent on experimental timing. This has been reported before (38) and suggests that ORF20 localization might be highly dynamic and only transiently needed at the replication compartment.

Replication compartments are enriched for many host and viral proteins involved in lytic DNA replication that shuttle in and out depending on their function during this process (35, 39). The formation of replication compartments is a complex but common process for viruses: these membraneless compartments grow and coalesce over infection, using liquid-liquid phase separation and pushing host chromatin to the nuclear periphery (40–42). Replication compartments are the site for viral genome replication, transcription, and packaging (19). Finding ORF20 at this site thus brings up an important question: is ORF20 involved in KSHV DNA replication? Past work on ORF20 orthologs has suggested that this viral protein is important for viral replication (43), yet the precise mechanism is still unclear. However, the ORF20 ortholog in MHV68 was not classified as an essential viral gene (44), suggesting that ORF20 contribution to DNA replication might vary slightly among the various herpesviruses.

One particularity of ORF20 is that it harbors a conserved PD-(D/E)XK endonuclease domain (45, 46). This signature nuclease domain is found in a number of proteins that can cleave nucleic acids. The other known KSHV PD-(D/E)XK nuclease is the viral protein SOX, which is responsible for host shutoff during the lytic phase (47–49). It is thus interesting to note that a second viral nuclease is harbored by KSHV, and since we are placing ORF20 in the replication compartment, this nuclease activity could be important for DNA replication. The DNase activity of SOX was previously hypothesized to be responsible for initiating DNA replication when a viral endonuclease is needed to create a nick in the origin of replication. However, this could never be proven, and instead, SOX DNase activity was shown to be important for branch resolution during replication (50). Similarly, in HSV-1, SOX ortholog UL12 was demonstrated to be involved in recombination (51) but has not yet been shown to be the nickase necessary for rolling circle replication initiation. This therefore leaves open the question of which protein—viral or cellular—carries out this single-stranded cleavage at the origin of replication during herpesviruses lytic replication. It would be interesting to test whether the ORF20 PD-(D/E)XK domain is functional and whether this viral protein contributes to this process.

Like that of other herpesviruses, KSHV lytic DNA replication is distinct from latent viral DNA replication, both because it uses a specific lytic origin of replication and because KSHV relies on viral and not cellular factors to regulate this process. Regulation of this process is complex and extensive; more work is necessary to fully understand this crucial viral mechanism. As we understand more and more about these previously uncharacterized viral proteins, such as ORF20, we are getting closer to this goal. Given the frequently multifunctional roles of viral proteins, it will be of interest to also explore possible additional activities of ORF20, including how it may contribute to nuclear remodeling during KSHV replication and whether ORF20 has an intrinsic nuclease activity.

## MATERIALS AND METHODS

### Cells and transfections.

HEK293T cells (ATCC) were grown in Dulbecco’s modified Eagle medium (DMEM; Invitrogen) supplemented with 10% fetal bovine serum (FBS). The KSHV-infected renal carcinoma human cell line iSLK.BAC16 (52) (kind gift from B. A. Glaunsinger) bearing doxycycline-inducible RTA was grown in DMEM supplemented with 10% FBS. For reactivation, BAC16-containing iSLK cells were treated with 1 μg/ml doxycycline and 1 mM sodium butyrate for the designated times. The KSHV-positive B cell line bearing a doxycycline-inducible version of the major lytic transactivator RTA (TREX-BCBL1) (53) was maintained in RPMI medium (Invitrogen) supplemented with 10% FBS (Invitrogen), 200 μM l-glutamine (Invitrogen), 100 U/ml penicillin-streptomycin (Invitrogen), and 50 μg/ml hygromycin B (Omega Scientific). Lytic reactivation was induced by treatment with 20 ng/ml 2-*O*-tetradecanoylphorbol-13-acetate (TPA; Sigma), 1 μg/ml doxycycline (BD Biosciences), and 500 ng/ml ionomycin (Fisher Scientific) for 48 h.

The KSHV ORF20 premature stop codon (ORF20_STOP_) mutant was engineered using the scarless Red recombination system in BAC16 GS1783 Escherichia coli as previously described (52), except using two gBlocks (IDT) to introduce the mutation. Each gBlock contained half of the kanamycin resistance cassette, as well as the desired mutation, and was joined by short overlap extension PCR before being used as the linear insert in the established protocol. The BAC16 ORF20 mutant was purified using the NucleoBond BAC 100 kit (Clontech). iSLK cell lines latently infected with the KSHV ORF20_STOP_ virus were then established by coculture; HEK293T cells were transfected with 5 μg of ORF20_STOP_ BAC DNA. The following day, the cells were trypsinized and mixed 1:1 with the KSHV-negative iSLK-puro cells and then treated with 25 nM 12-*O*-tetradecanoylphorbol-13-acetate (TPA) and 300 nM sodium butyrate for 4 days to induce lytic replication. iSLK cells were then selected using selection medium containing 300 μg/ml hygromycin B, 1 μg/ml puromycin, and 250 μg/ml G418. The medium was replaced every other day for ∼2 weeks, gradually increasing the hygromycin B concentration until there were no HEK293T cells remaining. These cells are here referred to as iSLK-ORF20_STOP_.

For DNA transfections, cells were plated and transfected after 24 h when 70% confluent using PolyJet (SignaGen).

Supernatant transfers were carried out in iSLK cells. The cells were reactivated for 48 h, and then the supernatants from induced iSLK cells were syringe filtered through a 0.45-μm-pore-size filter and spinfected into HEK293T cells at 1,500 rpm for 1.5 h at 37°C. Twenty-four hours later, cells were imaged on a fluorescence microscope.

### Plasmids.

The KSHV ORF20 open reading frame (ORF) was obtained from the KSHV ORFeome (54) and cloned into a pcDNA4 Nter-3×Flag vector. For the BirA-ORF20 construct, the sequence of the BirA ligase (containing a mutation [R118G] allowing it to act as a promiscuous biotin ligase) was PCR amplified from pcDNA3.1 MCS-BirA(R118G)-HA and cloned at the C terminus of ORF20 in the pcDNA4 Nter-3×Flag vector. All cloning steps were performed using in-fusion cloning (Clontech-TaKaRa) and were verified by Sanger sequencing.

### RT-qPCR.

Total RNA was harvested using TRIzol according to the manufacture’s protocol. cDNAs were synthesized from 1 μg of total RNA using avian myeloblastosis virus (AMV) reverse transcriptase (Promega) and used directly for quantitative PCR (qPCR) analysis with the SYBR green qPCR kit (Bio-Rad) on a QuantStudio 3 real-time PCR machine. Signals obtained by qPCR were normalized to those of 18S. For DNA replication calculation, iSLK cells were incubated with 5× proteinase K digestion buffer (50 mM Tris-HCl [pH 7.4], 500 mM NaCl, 5 mM EDTA, 2.5% SDS) and digested with proteinase K (80 μg/ml) overnight at 55°C. The genomic DNA (gDNA) was isolated by using a Zymo Quick gDNA miniprep kit according to the manufacturer’s instructions. DNA levels were quantified using relative standard curves with primers specific for KSHV *ORF59* (5′-AATCCACAGGCATGATTGC-3′ and 5′-CACACTTCCACCTCCCCTAA-3′) or a region in the *GAPDH* promoter (5′-TACTAGCGGTTTTACGGGCG-3′ and 5′-TCGAACAGGAGGAGCAGAGAGCGA-3′). The relative genome numbers were normalized to *GAPDH* to account for loading differences and to uninduced samples to account for the starting genome copy number.

### Western blotting.

Cell lysates were prepared in lysis buffer (150 mM NaCl, 50 mM Tris, 0.5% NP-40, 1 mM dithiothreitol [DTT], and protease inhibitor tablets) and quantified by Bradford assay. Equivalent amounts of each sample were resolved by SDS-PAGE and Western blotted with the following antibodies at 1:1,000 in TBST (Tris-buffered saline, 0.1% Tween 20): rabbit anti-ORF20 (Yenzym) rabbit anti-DHX9/RNA helicase A (Abcam), rabbit anti-glyceraldehyde-3-phosphate dehydrogenase (GAPDH) (Abcam), rabbit anti-vinculin (Abcam), and rabbit anti-HA (Abcam) antibodies. The rabbit anti-ORF59 and anti-K8.1 antibodies were used at 1:10,000 and 1:50,000, respectively, and were a gift from the Glaunsinger lab. Primary antibody incubations were followed by horseradish peroxidase (HRP)-conjugated goat anti-mouse or goat anti-rabbit secondary antibodies (Southern Biotechnology, 1:5,000).

### Immunoprecipitation.

Cells were lysed in low-salt lysis buffer (150 mM NaCl, 0.5% NP-40, 50 mM Tris [pH 8], 1 mM DTT, and protease inhibitor cocktail), and protein concentrations were determined by Bradford assay. Equivalent quantities of each sample and at least 400 μg of total protein were incubated overnight with the designated antibody and then with protein G-coupled magnetic beads (Life Technologies) for 1 h. Beads were washed extensively with lysis buffer. Samples were resuspended in Western blot loading buffer before resolution by SDS-PAGE.

### Immunofluorescence assays.

iSLK-ORF20_STOP_ or TREX-BCBL1 cells were grown on coverslips and fixed in 4% formaldehyde for 20 min at room temperature. Cells were then permeabilized in 1% Triton X-100 and 0.1% sodium citrate in phosphate-buffered saline (PBS) for 10 min, saturated in bovine serum albumin (BSA) for 30 min, and incubated with the designated antibodies at a 1:100 dilution. After 1 h, coverslips were washed in PBS and incubated with Alexa Fluor 594 or Alexa Fluor 488 secondary antibodies at 1:1,500 (Invitrogen). Coverslips were washed again in PBS and mounted in DAPI-containing Vectashield mounting medium (Vector Labs) to stain cell nuclei before visualization by confocal microscopy on a Nikon A1 resonant scanning confocal microscope (A1R-SIMe). The microscopy data were gathered in the Light Microscopy Facility and Nikon Center of Excellence at the Institute for Applied Life Sciences, UMass Amherst, with support from the Massachusetts Life Sciences Center.

### BioID and mass spectrometry.

BioID was originally developed by Roux et al. (55) and makes use of a promiscuous biotin ligase: the BirA ligase. Samples were prepared as in reference 34. Briefly, iSLK-ORF20_STOP_ cells were seeded in 10-cm plates and reactivated at the same time. Twenty-four hours later, cells were transfected using PolyJet with 5 μg of the ORF20-BirA construct (or mock-BirA control vector), and 50 μM Biotin was added to the medium. The following day, cells were harvested and lysed, and affinity purification was performed using magnetic streptavidin beads (Cell Signaling) overnight at 4°C. Beads were then extensively washed and trypsin digested overnight. Samples were then cleaned up using a C_18_ column, and mass spectral data were obtained at the University of Massachusetts Mass Spectrometry Center using an Orbitrap Fusion mass spectrometer.

### Statistical analysis.

All results are expressed as means ± standard errors of the means (SEMs) of experiments independently repeated at least three times. Unpaired Student’s *t* test was used to evaluate the statistical difference between samples. Significance was evaluated with *P* values and represented as follows: *, *P* < 0.05; **, *P* < 0.01; ***, *P* < 0.001; ns, nonsignificant.
