# Supplementary material for: The KSHV ORF20 Protein Interacts with the Viral Processivity Factor ORF59 and Promotes Viral Reactivation
Source: Microbiol Spectr. 2021 Jun 9;9(1):10.1128/spectrum.00145-21. doi: 10.1128/spectrum.00145-21 (PMC8552657; doi:10.1128/spectrum.00145-21)

**Supplementary Figure Legends**

**Supplementary Table 1: Complete list of proteins identified by BioID followed by mass spectrometry.** All proteins identified by MS after BioID proximity labeling, including accession number and peptide count. Each tab is a different biological replicate experiment and the last tab is the combined high confidence list. Proteins that were previously identified in Bussey *et. al.* are highlighted with a green font.

**Supplementary figure 1: Newly generated ORF20 antibody recognized ectopic ORF20-tagged expression.**


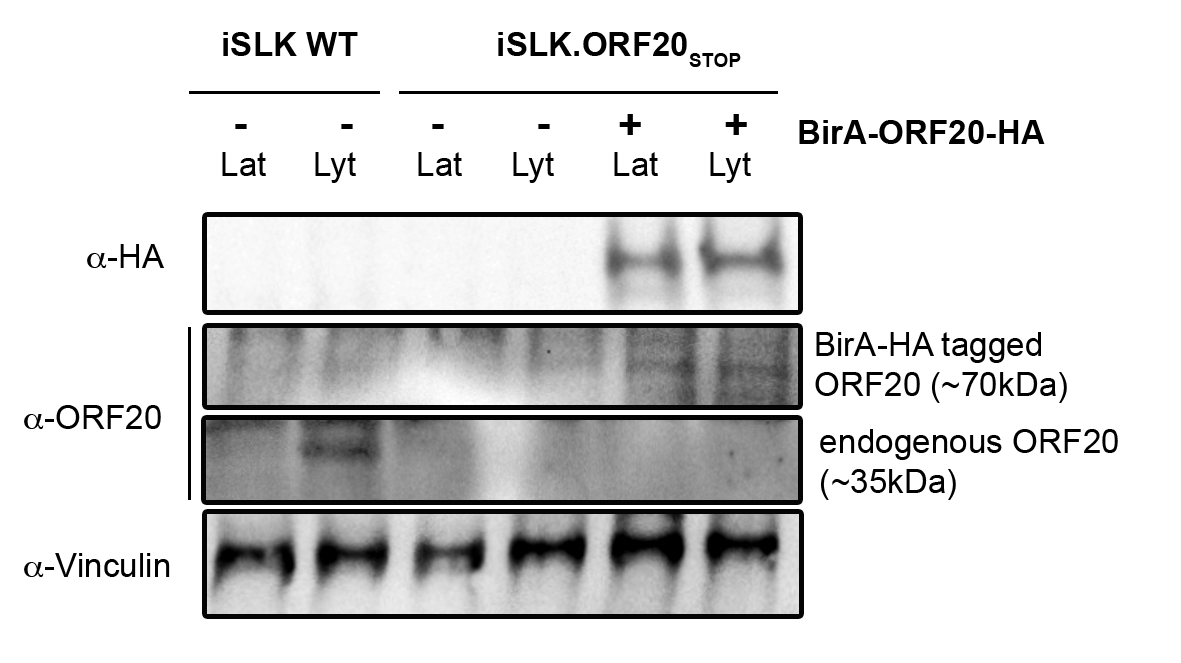

Supplement: SUPPLEMENTAL FILE 1 — FIG S1. Download SPECTRUM00145-21_Supp_1_seq2.docx, DOCX file, 0.2 MB [file spectrum00145-21_supp_1_seq2.docx]
